# KLC1-ROS1 Fusion Exerts Oncogenic Properties of Glioma Cells via Specific Activation of JAK-STAT Pathway

**DOI:** 10.3390/cancers16010009

**Published:** 2023-12-19

**Authors:** Takashi Fujii, Yoshiko Nakano, Daichi Hagita, Nobuyuki Onishi, Arumu Endo, Masaya Nakagawa, Toru Yoshiura, Yohei Otsuka, Satoru Takeuchi, Mario Suzuki, Yuzaburo Shimizu, Terushige Toyooka, Yuko Matsushita, Yuko Hibiya, Satoshi Tomura, Akihide Kondo, Kojiro Wada, Koichi Ichimura, Arata Tomiyama

**Affiliations:** 1 Department of Brain Disease Translational Research, Juntendo University Faculty of Medicine, 2-1-1 Hongo, Bunkyo-ku, Tokyo 113-8421, Japan; ta-fujii@juntendo.ac.jp (T.F.); d.hagita.on@juntendo.ac.jp (D.H.); y.matsushita.mc@juntendo.ac.jp (Y.M.); y.hibiya.ez@juntendo.ac.jp (Y.H.); k.ichimura.uk@juntendo.ac.jp (K.I.); 2 Department of Neurosurgery, National Defense Medical College, 3-2 Namiki, Tokorozawa 359-8513, Saitama, Japan; aendo@ndmc.ac.jp (A.E.); mnakagawa@ndmc.ac.jp (M.N.); tyoshiura@ndmc.ac.jp (T.Y.); yotsuka@ndmc.ac.jp (Y.O.); stake@ndmc.ac.jp (S.T.); toyo@ndmc.ac.jp (T.T.); stingray@ndmc.ac.jp (K.W.); 3 Department of Neurosurgery, Juntendo University School of Medicine, 2-1-1 Hongo, Bunkyo-ku, Tokyo 113-8421, Japan; marisuzu@juntendo.ac.jp (M.S.); yzshimiz@juntendo.ac.jp (Y.S.); knd-aki@juntendo.ac.jp (A.K.); 4 Department of Pediatrics, The University of Tokyo Hospital, 7-3-1 Hongo, Bunkyo-ku, Tokyo 113-8655, Japan; yonakano@g.ecc.u-tokyo.ac.jp; 5 Department of Clinical Diagnostic Oncology, Clinical Research Institute for Clinical Pharmacology and Therapeutics, Showa University, 1-5-8 Hatanodai, Shinagawa-ku, Tokyo 142-8555, Japan; nobuyukionishi@med.showa-u.ac.jp; 6 Division of Traumatology, Research Institute, National Defense Medical College, 3-2 Namiki, Tokorozawa 359-8513, Saitama, Japan; tomura@ndmc.ac.jp

**Keywords:** glioma, oncogene, KLC1-ROS1 fusion, JAK-STAT pathway

## Abstract

**Simple Summary:**

By examining the detailed molecular oncogenic mechanisms of KLC1-ROS1 fusion, the function of a novel RTK fusion was investigated to discover novel therapeutic targets for glioma. When wild-type *ROS1* and *KLC1-ROS1* fusions were expressed in human glioma cell lines, immunoblotting revealed that KLC1-ROS1 fusion specifically activated the JAK2–STAT3 axis compared with wild-type ROS1. Immunoprecipitation revealed a more efficient association of KLC1-ROS1 fusion with JAK2 compared with wild-type ROS1. A mutagenesis study of *KLC1-ROS1* fusion demonstrated the essential roles of both the KLC1 and ROS1 domains in the serum factor-independent constitutive activation of the fusion. In addition, KLC1-ROS1 fusion induced the upregulation of cell proliferation and invasion compared to wild-type ROS1 in a JAK-STAT-dependent manner under serum deprivation. Overall, our results suggested that molecules other than RTKs may serve as novel therapeutic targets for gliomas with RTK fusions.

**Abstract:**

Here, we investigated the detailed molecular oncogenic mechanisms of a novel receptor tyrosine kinase (RTK) fusion, *KLC1-ROS1*, with an adapter molecule, KLC1, and an RTK, ROS1, discovered in pediatric glioma, and we explored a novel therapeutic target for glioma that possesses oncogenic RTK fusion. When wild-type *ROS1* and *KLC1-ROS1* fusions were stably expressed in the human glioma cell lines A172 and U343MG, immunoblotting revealed that *KLC1-ROS1* fusion specifically activated the JAK2-STAT3 pathway, a major RTK downstream signaling pathway, when compared with wild-type *ROS1*. Immunoprecipitation of the fractionated cell lysates revealed a more abundant association of the KLC1-ROS1 fusion with JAK2 than that observed for wild-type ROS1 in the cytosolic fraction. A mutagenesis study of the KLC1-ROS1 fusion protein demonstrated the fundamental roles of both the KLC1 and ROS1 domains in the constitutive activation of KLC1-ROS1 fusion. Additionally, in vitro assays demonstrated that KLC1-ROS1 fusion upregulated cell proliferation, invasion, and chemoresistance when compared to wild-type ROS1. Combination treatment with the chemotherapeutic agent temozolomide and an inhibitor of ROS1, JAK2, or a downstream target of STAT3, demonstrated antitumor effects against KLC1-ROS1 fusion-expressing glioma cells. Our results demonstrate that KLC1-ROS1 fusion exerts oncogenic activity through serum-independent constitutive activation, resulting in specific activation of the JAK-STAT pathway. Our data suggested that molecules other than RTKs may serve as novel therapeutic targets for RTK fusion in gliomas.

## 1. Introduction

In recent years, oncogenic fusion genes have been identified in various cancer types because of the recent undertaking of extensive genomic analyses of clinical samples [1,2,3]. These oncogenic fusions may serve as potential therapeutic targets. In particular, fusion genes comprising the kinase domains of druggable receptor tyrosine kinases (RTKs), such as epidermal growth factor receptors or anaplastic lymphoma kinases, are used clinically as favorable therapeutic targets against malignancies [2,4,5,6]. Therefore, screening for oncogenic fusions is important for the diagnosis and treatment of cancers.

Gliomas are the most common and aggressive intracranial malignancies arising from brain parenchyma, and the prognosis is inadequate despite recent advances in their diagnosis and therapy [7,8,9]. In recent extensive genome analyses of glioblastomas (GBM), oncogenic fusions, particularly those involving the kinase domains of RTKs, have been discovered, and some of these fusions are expected to be novel therapeutic targets against GBMs [10,11].

We previously reported KLC1-ROS1, a novel fusion protein discovered in a pediatric glioma patient, and demonstrated its potential as an oncogene [12]. This fusion is composed of the N-terminal region of a member of the kinesin light chain family, kinesin light chain 1 (KLC1), which associates with cytoplasmic cargo as well as kinesins and shuttles them along with cytoplasmic microtubules, and the C’-C-terminal kinase domain of RTK, ROS1. ROS1 is a member of the RTKs family, in which the kinase domain possesses considerable homology with other RTKs, particularly with anaplastic lymphoma kinase [13,14]. Although an association of neural epidermal growth factor–like 2N’-terminal domain (NELL2), a lumicrine factor secreted from testicular germ cell, with the N’-terminal extracellular domain of ROS1 is suggested in a murine system [15], the ligand(s), activation machinery, and biological roles of wild-type ROS1 in humans remain unclear [16,17]. Previous evidence has shown that wild-type ROS1 has the potential to trigger the activation of downstream RTK signaling, that is, the PI3K-mTOR, Jak-STAT, and RAS-MAPK pathways, thereby inducing cellular proliferation, migration/invasion, and survival [18,19]. Contrary to the wild-type ROS1, oncogenic fusion composed of the C’-terminal activation loop of ROS1 has already been discovered in various neoplasms, while there are several earlier reports in the case of GBM [12,18,20,21,22,23]. Targeting the C-terminal activation loop of ROS1 in the treatment of ROS1-fusion-positive tumors using small-molecule inhibitors of ROS1 has been conducted; however, the adverse effects of these ROS1 inhibitors have hindered their clinical use [18,24]. Particularly in the case of brain tumor treatment, the high-dose systemic administration of each drug is necessary to achieve higher concentrations in the brain tissue by penetrating the blood–brain barrier (BBB). Therefore, to treat ROS1-fusion-positive brain tumors more effectively and safely, exploring novel therapeutic target(s) of ROS1-fusion-positive tumors other than the C-terminal activation loop of ROS1 is warranted.

In this study, we investigated the detailed molecular signaling pathway triggered by KLC1-ROS1 fusion in glioma cells and discovered that JAK-STAT signaling was specifically activated in KLC1-ROS1 fusion-expressing human glioma cells. The inhibition of JAK-STAT signaling successfully suppressed the oncogenic properties of KLC1-ROS1 fusion-expressing human glioma cells, suggesting that JAK-STAT signaling could be a novel therapeutic target against KLC1-ROS1 fusion-positive gliomas.

## 2. Materials and Methods

### 2.1. Reagents and Antibodies

RPMI 1640 medium (#11875176), 10% fetal bovine serum (FBS), 1% penicillin–streptomycin (#15140122), Hoechst 33342 (Ho, #B2261), Propidium Iodide (PI, #P1304MP), Lipofectamine^®^ 3000 Reagent (#L3000008), Lipofectamine RNAiMAX (#13778075), bismaleimidohexane (BMH, #22330), and PIERCE assay kit were purchased from Thermo Fisher Scientific (Tokyo, Japan). The protease inhibitor cocktail (#25955-11) and EDTA-free phosphatase inhibitor cocktail (07575-51) were purchased from Nacalai Tesque, Inc. (Kyoto, Japan). Ruxolitinib (#S1378), baricitinib (#S2851), crizotinib (#S1068), sabutoclax (#), and lorlatinib (#7536) were purchased from Selleck (Tokyo, Japan). Western Lightning Plus-ECL (#NEL1015001EA) was purchased from PerkinElmer Japan (Yokohama, Japan). The Can Get Signal was purchased from TOYOBO (Osaka, Japan). Primary antibodies against beta-Actin (#5125), phospho-Akt (Ser 473) (#9271,), total Akt (#9272), c, phospho-Erk 1/2 (Thr 202/Tyr 204) (#9101), total Erk 1/2 (#9102), GAPDH (3683), total JAK2 (#3230), pohspho-JAK2 (Y1007/1008) (#3776), Mcl-1 (#5453), c-Myc (#5605), matrix metalloproteinases 2 (MMP2) (#40994), Poly (ADP-ribose) polymerase (PARP) (#9542), phospho-ROS1 (Y2274/) (#3078), total ROS1 (#3266), phospho-signal transducer and activator of transcription 3 (STAT3, Tyr 705) (#9145), total STAT3 (#9139), alpha-Tubulin (#2125), and HRP-conjugated anti-rabbit secondary antibody (#7074) were purchased from Cell Signaling Technology (Tokyo, Japan). Peroxidase-conjugated anti FLAG M2 monoclonal antibody (#A8592) and FLAG M2 monoclonal antibody affinity gel (#A2220) were obtained from MERK (Tokyo, Japan). Matrigel^®^ Matrix (#354230) was purchased from CORNING (Tokyo, Japan). Temozolomide (TMZ) was from FUJIFILM Wako (Osaka, Japan).

### 2.2. Cell Culture

Human GBM cell lines A172 and U343MG were obtained from the American Type Culture Collection (Manassas, VA, USA). These cells were maintained as an adherent monolayer by serial passage at 37 °C under 5% CO_2_ conditions and cultured by RPMI 1640 medium supplemented with 10% FBS and 1% penicillin–streptomycin.

### 2.3. Plasmids

Plasmids encoding *wild-type ROS1* (*ROS1* isoform 1) were synthesized using GenScript Japan (Tokyo, Japan). *KLC1-ROS1* fusion and mutants of the *KLC1-ROS1* fusion (fusion with *ROS1* kinase dead point mutation (K1980M] and fusion with *KLC1* domain deletion) were obtained as previously described [12]. These constructs were subcloned into the pPCIP piggybac (PB) system [25] for transfection. An expression vector containing the hyperactive PB transposase was constructed as previously described [26].

### 2.4. Plasmid Transfection

For transient transfection experiments of plasmids, the pPCIP-based plasmids described above (3 μg) were transfected into glioma cells cultured in 6-well plates using Lipofectamine^®^ 3000 Reagent. Establishment of stable transfectant was performed by co-transfection of pPCIP-based plasmids with the plasmid-encoding PB transposase (pPCIP:transposase = 4:1) using Lipofectamine^®^ 3000 Reagent. After 24 h, the cells were treated with 4 μg/mL puromycin for 72 h to select the stable transfectants.

### 2.5. Cell Lysates Preparation, Subcellular Fractionation, In Vitro Protein Crosslinking, and Immunoblotting

Harvested cells were washed with PBS and lysed with lysis buffer A (50 mM Tris-HCl pH 7.2, 150 mM NaCl, 0.1% SDS, 1% deoxycholate, 1% Triton X 100), then supplemented with protease inhibitor cocktail and phosphatase inhibitor cocktails on ice for 30 min with continuous shaking. After removing debris by centrifugation, the supernatants were collected as whole-cell lysates. The protein concentration of each cell lysate was determined using a PIERCE assay kit. Immunoblotting was performed as previously described [27] with modifications. The antibodies were diluted in Can Get Signal^®^, and the final chemiluminescence signals were illuminated by Western Lightning Plus-ECL and captured using Amersham Imager 600 (GE Healthcare, Chicago, IL, USA). Subcellular fractionation of cell lysates was performed using a Minute Plasma Membrane Protein Isolation Kit (Invent Biotechnologies, Plymouth, MN, USA). The nuclear fraction was extracted by lysing the nuclear pellet of each sample obtained using the Minute Plasma Membrane Protein Isolation Kit with buffer A supplemented with a protease inhibitor cocktail and phosphatase inhibitor cocktail. Immunoblotting combined with in vitro protein crosslinking was performed as previously described, using the protein crosslinker bismaleimidohexane (BMH) [28] with modifications. Briefly, after harvest, the cells were treated with 5 mM BMH for 30 min at 25 °C with continuous rocking for in vitro protein crosslinking. After quenching the crosslinking reaction by adding glycine, cells were lysed using lysis buffer as described above, and cell lysates were analyzed by immunoblotting.

### 2.6. Immunoprecipitation

Immunoprecipitation of cell lysates was performed as previously described [29] using a FLAG M2 monoclonal antibody affinity gel. Total 1000 μg/protein of whole cell lysates or 100 μg/protein of cytosolic cell lysates per sample was immunoprecipitated by 10 μL of FLAG M2 monoclonal antibody affinity gel.

### 2.7. Total Cell Count Assay

Cells (1 × 10^4^) were plated in 6-well dishes and grown for the indicated times with or without treatment. The cells in each well were collected in 15 mL tubes and pelleted by centrifugation. The pelleted cells were resuspended in 1 mL PBS, and the number of cells in the cell suspension was counted using Countess^TM^ 2 (Thermo Fisher Scientific, Tokyo, Japan).

### 2.8. Cell Death Assay

The cell death assay was done as previously described [28]. Totals of 1 × 10^5^ cells/sample were seeded on 6-well plates. After 24 h, the cells were treated with TMZ alone, or TMZ with 2 h pretreatment by vehicle (DMSO) or small molecule inhibitors as indicated. The cells were then further cultured for 72 h and co–stained with Ho and PI. The rate of the PI positive cells (dead cells) to Ho positive cells (total cells) was calculated as the cell death rate. Fluorescence images of stained cells were obtained using a BZ-X700 microscope (KEYENCE, Osaka, Japan).

### 2.9. Transwell Invasion Assay

Transwell invasion assays using 8 μm polycarbonate Transwell filter chambers (#353097; Corning, Corning, NY, USA) in 24-well plates were performed as previously described [30], with minor modifications. The top surfaces of the Transwell membranes were coated with Matrigel^®^ Matrix. The cells were seeded at 50,000 cells·well^−1^ into the top chambers of the Transwell plates. To minimize the effect of cell proliferation, the cells were fixed12 h after being seeded in the upper chamber.

### 2.10. Gelatin Zymogram

Gelatin zymogram was done as previously described [27].

### 2.11. Statistical Analysis

All quantitative results are demonstrated as the average ± standard deviations of three independent experiments and were analyzed by unpaired Student’s *t*-test using Microsoft 365 Excel. Statistical significance was set at: *p* values < 0.01 were considered significant.

## 3. Results

### 3.1. KLC1-ROS1 Fusion Specifically Activated JAK-STAT Pathway

**Figure 1:**
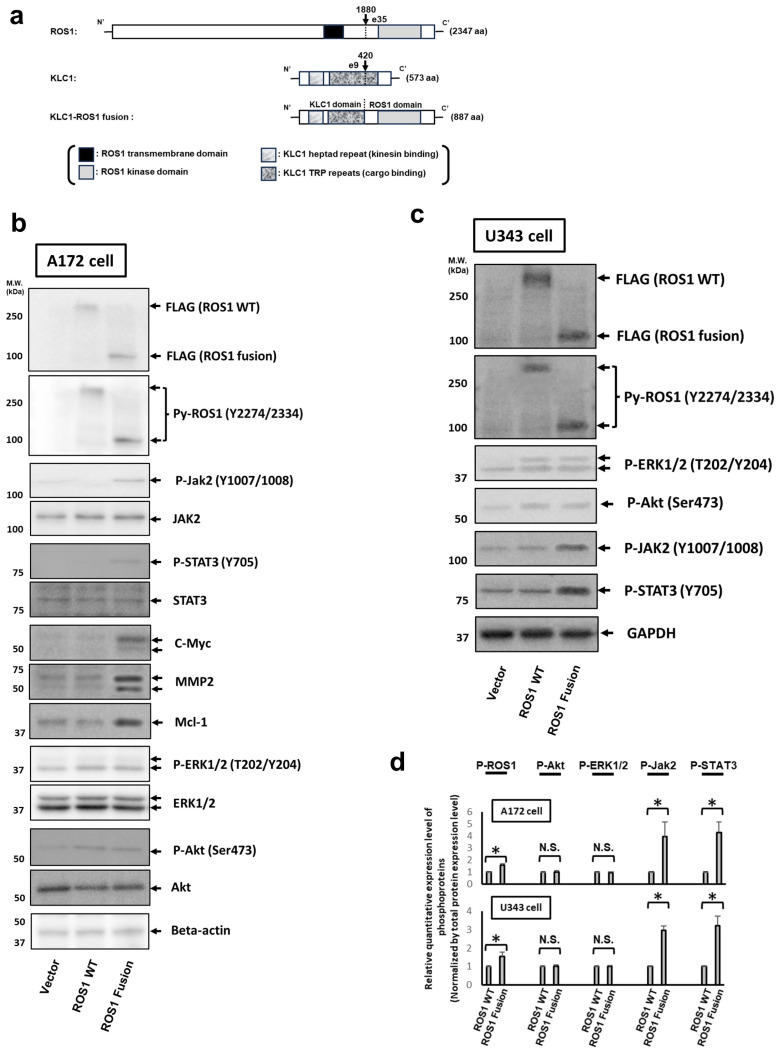
Expression of KLC1-ROS1 fusion specifically activates the JAK-STAT pathway in glioma cells. (**a**) Schematic illustration of the domain structure of wild-type ROS1 (ROS1), wild-type KLC1 (KLC1), and KLC1-ROS1 fusion. The breakpoints of ROS1 (exon35) and KC1 (exon9) are indicated by an arrowhead with the corresponding amino acid (aa) positions. (**b**,**c**) Plasmids encoding the empty vector, *FLAG*-tagged *wild-type* (WT) *ROS1*, and *FLAG*-tagged *KLC1-ROS1* fusion were stably expressed in A172 and U343MG human glioblastoma cell lines ((**b**) A172 cells, (**c**) U343MG cells). The cell lysates were analyzed by immunoblotting using the indicated antibodies. Primary anti-beta actin antibody and anti-GAPDH antibodies were used for confirming the protein loading amount of each sample. The uncropped blots are shown in File S1. (**c**) Three sets of A172 and U343MG cells stably expressing *FLAG*-tagged *WT ROS1* and *FLAG*-tagged *KLC1-ROS1* fusion proteins were established independently, and their cell lysates were analyzed by immunoblotting as shown in Figure 1b,c. (**d**) Changes in FLAG-tagged WT ROS1, FLAG-tagged KLC1-ROS1 fusion, Akt, ERK1/2, JAK2, and STAT3 activities after expressing *FLAG*-tagged *ROS1* constructs in each cell were quantified by immunoblotting and calculated as follows: (phospho-protein values)/(FLAG-tagged WT ROS1 or FLAG-tagged KLC1-ROS1 fusion values). The mean of the calculated relative activities (value of WT ROS1-expressing cell = 1) is presented as a bar graph. * *p* > 0.01. N.S.: No Significance.

Since our previous report clarified that KLC1-ROS1 fusion is composed of the C’-terminal ROS1 domain and the N’-terminal KLC1 domain, which possesses both the kinase domain of wild-type ROS1 and the kinesin/cargo binding domains of wild-type KLC (Figure 1a) [12], we first hypothesized that the active center of KLC1-ROS1 fusion might be the ROS1 kinase domain. Therefore, to investigate the oncogenic roles of KLC1-ROS1 fusion in glioma cells, plasmids encoding C’-terminal FLAG-tagged wild-type ROS1 and KLC1-ROS1 fusion as well as the empty vector were stably expressed in the A172 GBM cell line, which did not express endogenous wild-type ROS1 [12] and has already been used as the manipulable model cell in our previous study regarding KLC1-ROS1 fusion [12]. The phosphorylation of two tyrosine residues of the ROS1 activation loop (Tyr 2274/2334 of the wild-type ROS1), which reflects activation of the ROS1 kinase domain, and major RTK downstream signaling pathways, namely the Akt-mTOR, MEK-ERK, and JAK-STAT pathways [8], was analyzed by the immunoblotting of each cell lysate (Figure 1b). While the expressions of FLAG-tagged wild-type ROS1 and KLC1-ROS1 fusion were almost equal, the phosphorylation (activation) of KLC1-ROS1 fusion was upregulated when compared with wild-type ROS1 (156 ± 11% of wild-type ROS1) (Figure 1b). However, the specific upregulation of JAK2 (Tyr1007/1008) and STAT3 (Tyr705) phosphorylation, which reflect the activation of JAK2 and STAT3 [31,32], respectively, was observed only in KLC1-ROS1 fusion-expressing cells when compared with wild-type ROS1 expressed cells (393 ± 121% and 429 ± 87% of wild-type ROS1 in JAK2 and STAT3 activation, respectively) (Figure 1b). In addition, the specific upregulation of the expression of known oncogenic transcriptional target molecules of STAT3, namely cell cycle accelerator c-Myc, antiapoptotic bcl-2 family protein Mcl-1, and extracellular matrix degradation enzyme matrix metalloproproteinase-2 (MMP-2), in KLC1-ROS1 fusion-expressing cells when compared to wild-type ROS1 expressed cells was also confirmed by the immunoblotting of each cell lysate (Figure 1b). Similar results were obtained in the U343MG human glioma cell line, in which endogenous ROS1 expression and activation were undetectable (Figure 1c). To confirm these results, we established three independent sets of stable transfectants for both cell lines and analyzed their cell lysates by immunoblotting as shown in Figure 1b,c. Quantitative immunoblotting analysis revealed that JAK2 and STAT3 activities were markedly upregulated in KLC1-ROS1 fusion-expressing cells when compared with wild-type ROS1 expressed cells, whereas Akt and ERK1/2 activities were not (Figure 1c). In addition, only a slight increase in the activation of KLC1-ROS1 fusion was confirmed compared with that of wild-type ROS1 in both glioma cell lines (Figure 1d). Together with these results, the possible role of KLC1-ROS1 fusion as an oncogene via specific activation of the JAK-STAT pathway triggered by the ROS1 kinase domain activation-dependent machinery in multiple glioma cells was suggested.

### 3.2. Distinct Cytosolic Localization of KLC1-ROS1 Fusion with Wild-Type ROS1 Might Contribute to Activation of JAK-STAT Pathway by KLC1-ROS1 Fusion in Glioma Cell

**Figure 2:**
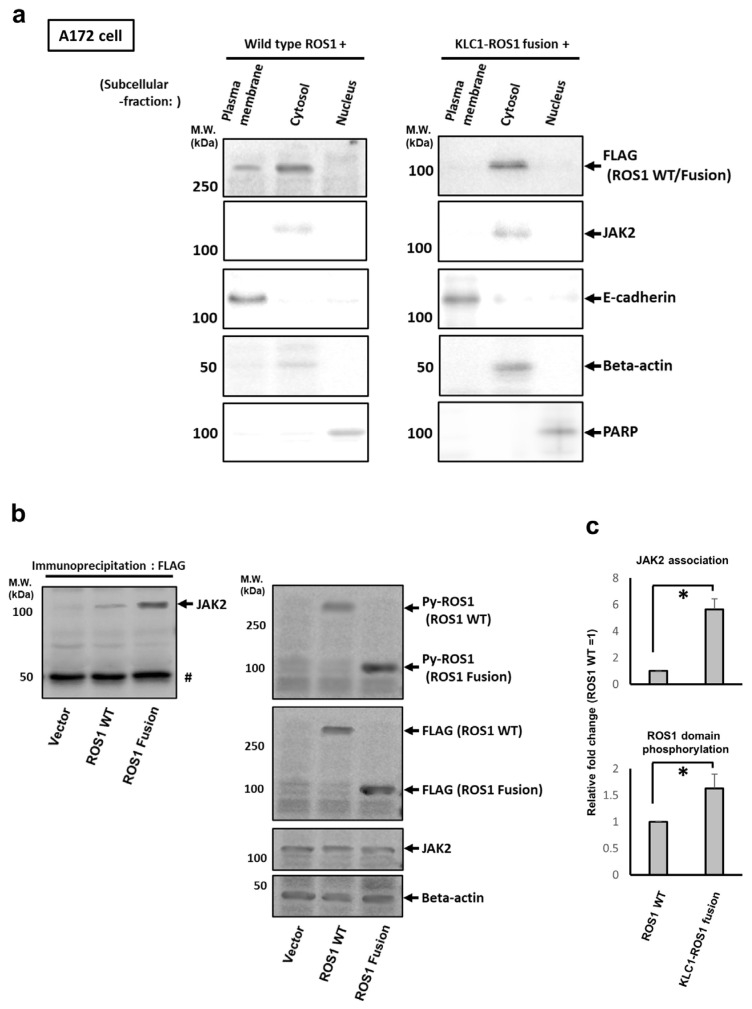
The KLC1-ROS1 fusion is specifically localized in cytoplasm and associates with JAK2. (**a**) *FLAG*-tagged *wild-type* (WT) ROS1 and *FLAG*-tagged *KLC1-ROS1* fusion were stably expressed in A172 cells. After 72 h, the cell lysates were fractionated into plasma membrane, cytosolic, and nuclear fractions. Fractionated cell lysates were analyzed by immunoblotting using the indicated primary antibodies. Primary antibody for e-cadherin, beta actin, and PARP were used as the fraction marker for plasma membrane, cytosol, and nucleus, respectively. # precipitated IgG. (**b**) (**Left**) The cytosolic fractions of cell lysates obtained in Figure 2a were subjected to immunoprecipitation using anti-FLAG M2 affinity gel, and the precipitates were analyzed by immunoblotting using a primary anti-JAK2 antibody. (**Right**) The input cytosolic fractions were analyzed by immunoblotting using the indicated primary antibodies. Primary anti-beta actin antibody was used for controlling the amount of protein loading in each sample. The uncropped blots are shown in File S1. (**c**) The ratio of JAK2 association efficiency and the ROS1 domain phosphorylation in WT ROS1 and KLC1-ROS1 fusion were calculated from the quantitative results of Figure 2b. The ratio of JAK2 association efficiency was calculated as follows: (WT ROS1 or KLC1-ROS1 fusion-bound JAK2 value (Figure 2b, **left**))/(total WT ROS1 or KLC1-ROS1 fusion value (Figure 2b, **right**)). The ratio of ROS1 domain phosphorylation was calculated as follows: (Py-WT ROS1 or KLC1-ROS1 fusion value (Figure 2b, **right**))/(total WT ROS1 or KLC1-ROS1 fusion value (Figure 2b, **right**)). These ratios were expressed as relative values (WT ROS1 = 1) by the graphs. * *p* > 0.01.

Next, the molecular mechanism by which KLC1-ROS1 fusion triggers JAK-STAT pathway activation in GBM cells was investigated. To clarify this, the subcellular localization of KLC1-ROS1 fusion was investigated first and then compared with that of wild-type ROS1 in GBM cells. C’-terminal FLAG-tagged wild-type ROS1 and FLAG-tagged KLC1-ROS1 were stably expressed in A172 GBM cells, as shown in Figure 1, and immunoblot analysis of fractionated cell lysates (the lysates of plasma membrane, total cytosol, and nucleus) of these cells demonstrated that KLC1-ROS1 fusion was specifically localized in cytosol, while wild-type ROS1 was localized in both the plasma membrane and cytoplasm, but was rather enriched in cytoplasm. (Figure 2a). Immunoblotting of immunoprecipitated cytosolic cell lysates of these cells using an anti-FLAG antibody showed that KLC1-ROS1 fusion is associated with JAK2 more abundantly than wild-type ROS1 (Figure 2b,c). In addition, the KLC1-ROS1/WT-ROS1 ratio of JAK2 association efficiency (5.64 ± 0.79/1) was found to be higher than the KLC1-ROS1/WT-ROS1 ratio of ROS1 domain phosphorylation (1.63 ± 0.27/1) (Figure 2b,c). These results suggest a possible mechanism that induces the ROS1 domain activation (phosphorylation)-independent association of KLC1-ROS1 fusion with JAK2 in the cytosol, resulting in a more efficient association of KLC1-ROS1 fusion with JAK2 than that of wild-type ROS1.

### 3.3. Both KLC1 Domain and ROS1 Domain Are Essential for Activation of KLC1-ROS1 Fusion as Well as Its Association with JAK2 in Glioma Cell

**Figure 3:**
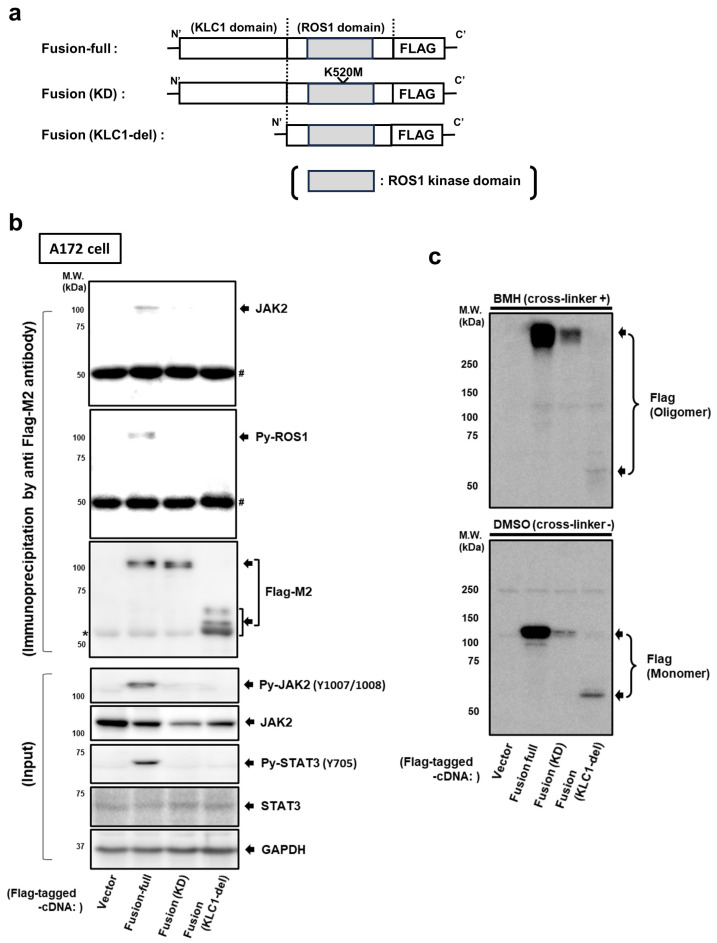
Both the KLC1 domain and ROS1 domain are essential for activation of KLC1-ROS1 fusion. (**a**) Schematic illustration of *FLAG*-tagged *KLC1-ROS1* fusion with or without mutation used in this study; C’-terminal *FLAG*-tagged *KLC1-ROS1* fusion without mutation (fusion-full); the *FLAG*-tagged *KLC1-ROS1* fusion with kinase dead point mutation of *ROS1* (*K520M*, fusion (KD)); *FLAG*-tagged *KLC1-ROS1* fusion with deletion of *KLC1* domain (fusion (KLC1-del)). (**b**) The full construct and mutants of *KLC1-ROS1* fusion protein in Figure 3a were transiently expressed in A172 cells. After 72 h, cell lysates were subjected to immunoprecipitation using a FLAG M2 affinity gel. Precipitates and input cell lysates were analyzed by immunoblotting using the indicated primary antibodies. The primary GAPDH antibody was used to control the amount of protein loading in each sample; * nonspecific bands; # precipitated IgG. (**c**) The mutants of *KLC1-ROS1* fusion mutants generated in Figure 2a were transiently expressed in A172 cells. After 72 h, the cells were treated with vehicle (DMSO) or cell-permeable protein crosslinker (BMH) for in vitro protein crosslinking (see Section 2), and these crosslinked cell lysates were analyzed by immunoblotting using the indicated primary antibodies. The uncropped blots are shown in File S1.

To further investigate how KLC1-ROS1 fusion is activated and associated with JAK2 by a molecular machinery different from that of wild-type ROS1, we next examined the role of the KLC1 and ROS1 domains of KLC1-ROS1 fusion in binding to and activating JAK2. The mutants of C’-terminal FLAG-tagged KLC1-ROS1 fusion, FLAG-tagged KLC1-ROS1 fusion lacking the KLC1 domain (KLC1-del), and FLAG-tagged KLC1-ROS1 fusion with kinase dead point mutation of the ROS1 kinase domain (ROS1-KD), which were used in our previous report (Figure 3a) [12], as well as KLC1-ROS1 fusion without mutation and an empty vector, were transiently expressed in A172 cells, and the activation of JAK2 and STAT3 in each cell was monitored by immunoblotting. Although enhanced activation of both JAK2 and STAT3 was confirmed in cells expressing KLC1-ROS1 fusion without mutation, the activation statuses of both JAK2 and STAT3 were not significantly changed in either the KLC1-del or ROS1-KD expressed cells compared with empty vector-expressing cells (Figure 3b). In addition, immunoblot analysis of immunoprecipitates of each cell lysate using an anti-FLAG antibody revealed that the KLC1-ROS1 fusion protein not only triggered tyrosine phosphorylation (activation) of its ROS1 kinase domain but was also associated with JAK2; however, neither the KLC1-del nor ROS1-KD proteins induced activation of their ROS1 kinase domain nor interacted with JAK2 (Figure 3b). These results suggest that both the KLC1 and ROS1 kinase domains of KLC1-ROS1 fusion are essential for the activation of the ROS1 kinase domain of KLC1-ROS1 fusion, which triggers JAK2-STAT3 signaling activation.

### 3.4. KLC1 Domain Rather Than ROS1 Domain Is Essential for Self-Oligomerization of KLC1-ROS1 Fusion

Since RTKs are known to form oligomers upon ligand-mediated activation [8,33], we hypothesized that KLC1-ROS1 fusion forms a ligand-independent oligomer, thereby inducing the hyperactivation of downstream signaling. To test this, C’-terminal FLAG-tagged KLC1-ROS1 fusion and its mutants described above, as well as an empty vector, were transiently expressed in A172 cells, and each cell lysate pretreated with a protein crosslinker (see Section 2) was analyzed by immunoblotting using anti-FLAG antibody. As a result, in KLC1-ROS1 fusion and ROS1-KD-expressing cell lysates, FLAG-tagged proteins appeared as a higher molecular weight mass (formation of multimer) in response to pretreatment with a protein crosslinker compared to pretreatment with vehicle (DMSO) (Figure 3b). Conversely, the same analysis of KLC1-del expressed cell lysates did not demonstrate a higher molecular weight mass, and only the original size mass was confirmed (Figure 3c). Together, these results suggest an essential role of the KLC1 domain in KLC1-ROS1 fusion in oligomerization, which is required for ROS1 kinase domain activation.

### 3.5. Ligand Independent Constitutive Activation of KLC1-ROS1 Fusion May Contribute to Its Oncogenic Machinery

**Figure 4:**
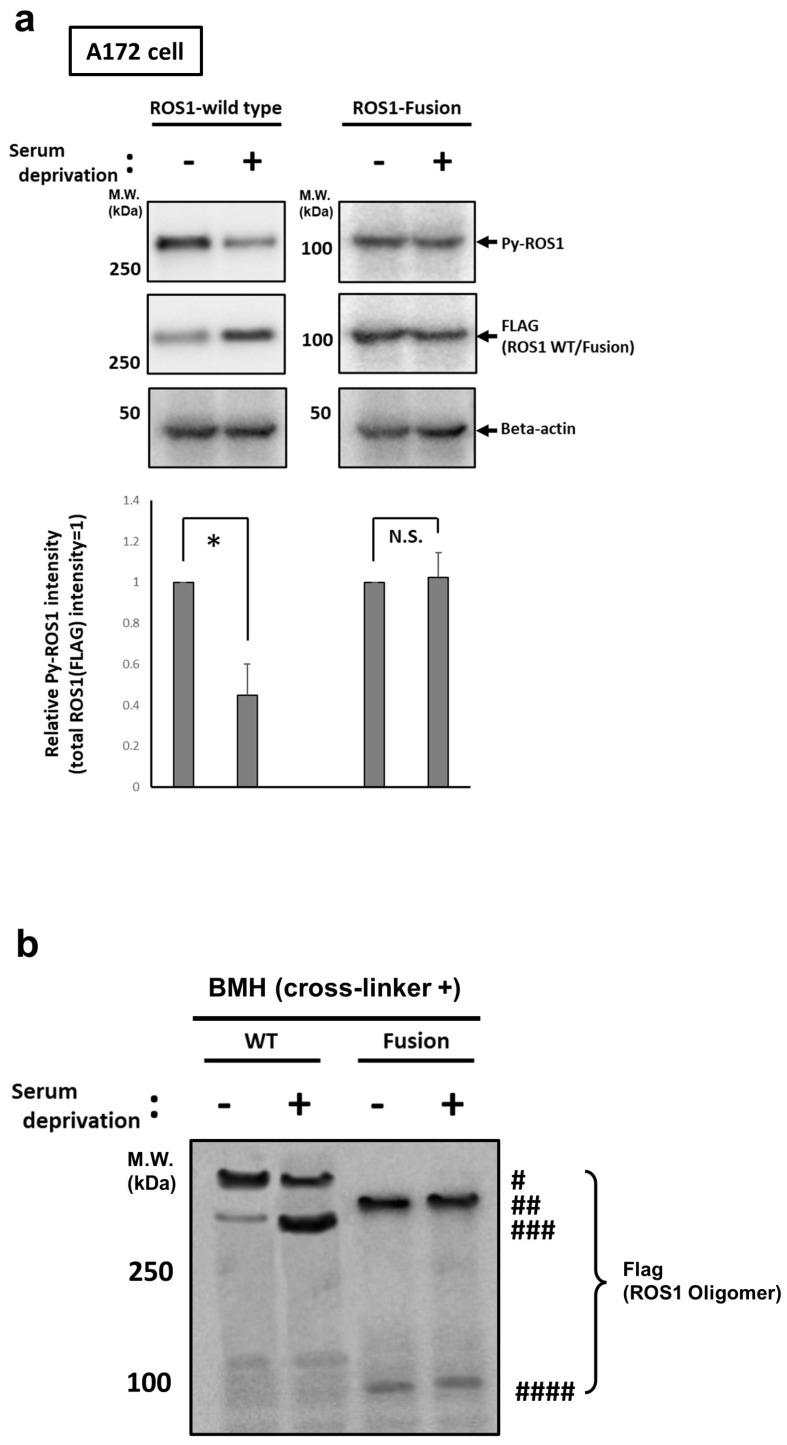
Serum-independent activation of KLC1-ROS1 fusion in glioma cells. (**a**) (**Top**) A172 cells, stably expressed FLAG-tagged wild-type (WT) ROS1, and FLAG-tagged KLC1-ROS1 fusion were cultured under serum-reduced condition (0.5%). After 12 h, these cell lysates were analyzed by immunoblotting using indicated primary antibodies. Primary beta actin antibody was used for confirming the protein loading amount of each sample. * *p* > 0.01. N.S.: No Significance. (**Bottom**) The relative phosphorylated ROS1 level in each treated cell was calculated by quantitative results of Figure 4a (phospho-ROS1 value/total ROS1 value). (**b**) A172 cells, stably expressed FLAG-tagged WT ROS1, and FLAG-tagged KLC1-ROS1 fusion were treated as shown in Figure 4a. After 12 h, these cells were subjected to in vitro protein crosslinking assay as shown in Figure 3c, and obtained crosslinked cell lysates were analyzed by immunoblotting using anti-FLAG antibody; # ROS1 WT multimer; ## ROS1 fusion multimer; ### ROS1 WT monomer; #### ROS1 fusion monomer. The uncropped blots are shown in File S1.

In most cases, after association with specific extracellular ligands (s), wild-type RTKs are immediately internalized into the cytosol from the plasma membrane via endocytosis and undergo proteasomal/lysosomal degradation or recycling to the plasma membrane [8,34]. To further explore the ligand-independent oncogenic activation of KLC1-ROS1 fusion, the alteration of activation and expression levels of both wild-type ROS1 and KLC1-ROS1 fusion after serum-reduced culturing was monitored. A172 cells stably expressing C’-C-terminal FLAG-tagged wild-type ROS1 and FLAG-tagged KLC1-ROS1 fusion were cultured for 24 h in a normal cell culture medium containing 10% serum (pre-serum deprivation) and then additionally cultured in serum-reduced media (0.5%) for 12 h (post serum deprivation). The cell lysates of these cells under pre-/post-serum deprivation were then analyzed by immunoblotting for the detection of tyrosine phosphorylation (=activation) and expression of FLAG-tagged wild-type ROS1 and FLAG-tagged KLC1-ROS1 fusion. These cells were further subjected to an in vitro protein crosslinking assay, followed by immunoblotting of their cell lysates to investigate their self-oligomerization (activation). In wild-type ROS1 expressed cells, the activation (tyrosine phosphorylation and formation of multimers) of wild-type ROS1 was suppressed post-serum deprivation compared to pre-serum deprivation (Figure 4a,b), whereas the expression level of total ROS1 was upregulated under the same conditions (Figure 4a,b). In the meantime, neither the activation (tyrosine phosphorylation and multimer formation) nor the expression of KLC1-ROS1 fusion were significantly altered pre-/post-serum deprivation compared with those of wild-type ROS1 (Figure 4a,b). These results suggest that KLC1-ROS1 fusion is continuously activated in the cytosol, regardless of the serum stimulation.

### 3.6. KLC1-ROS1 Fusion Results in Upregulated Glioma Cell Proliferation, Chemoresistance, and Invasion Compared with Wild-Type ROS1

**Figure 5:**
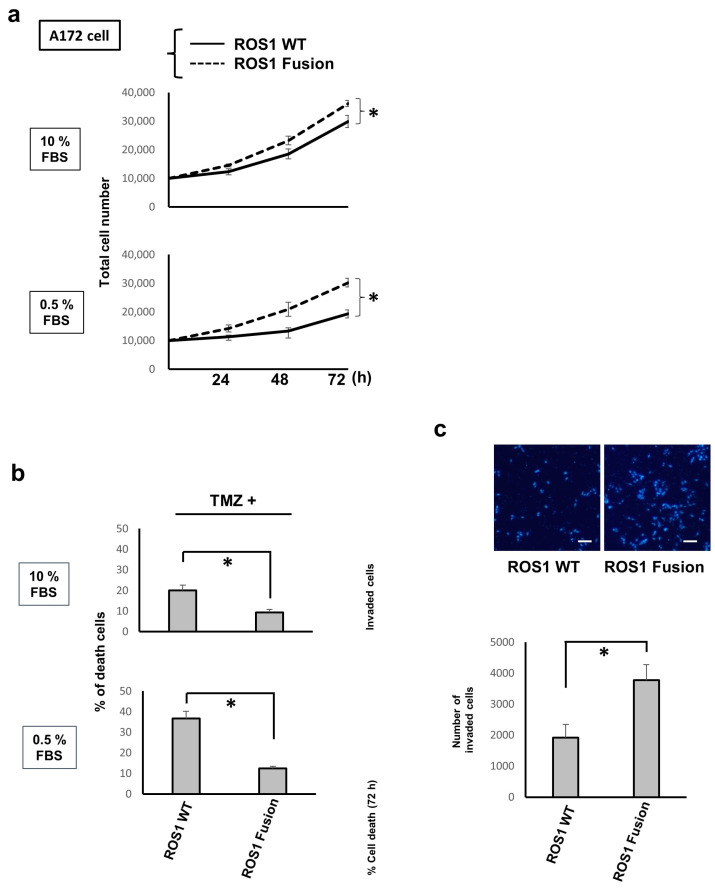
KLC1-ROS1 fusion induces enhanced oncogenic properties compared with wild-type ROS1 in fusion in glioma cells. (**a**) A172 cells stably expressing FLAG-tagged wild-type (WT) ROS1 and FLAG-tagged KLC1-ROS1 fusion, as seen in Figure 1a, were cultured in serum (10% FBS, **upper**) or serum-reduced conditions (0.5% FBS, **lower**). At the indicated time points, the total cell number in each cell culture was quantitated using a cell counting assay. * *p* > 0.01. (**b**) The A172 cells used in Figure 5a were cultured under treatment with Temozolomide (TMZ treatment. 150 μM) in serum or serum-reduced conditions, the same as seen in Figure 5a. After 72 h, cells were subjected to a cell death assay. * *p* > 0.01. (**c**) The A172 cells shown in Figure 5a were subjected to a cell invasion assay. Fluorescence microscopy images of the stained nuclei of invaded cells (bar, 50 μm) (**upper**) and the quantitative results of the invasion assay (**lower**) are shown. * *p* > 0.01.

Next, the regulation of the oncogenic phenotypes of glioma cells triggered by KLC1-ROS1 fusion was investigated. As representative oncogenic properties, the cell proliferation, invasion, and chemoresistance [35,36] of A172 cells stably expressing the C’-terminal FLAG-tagged wild-type ROS1 and FLAG-tagged KLC1-ROS1 fusion, as seen in Figure 1a, were investigated. As shown in Figure 5a,b, the proliferation and chemoresistance to TMZ, a first-line chemotherapeutic agent for GBMs, of KLC1-ROS1 fusion-expressing cells were upregulated when compared to wild-type ROS1-expressing cells, especially under serum-reduced conditions. Transwell-based cell invasion assays revealed an increased invasiveness of KLC1-ROS1 fusion-expressing cells when compared to wild-type ROS1-expressing cells (Figure 5c). From these results, an enhanced oncogenic function of the KLC1-ROS1 fusion in glioma cells compared to wild-type ROS1, especially under serum factor-deprived conditions, can be suggested.

### 3.7. Elevated Oncogenic Properties of Glioma Cells Induced KLC1-ROS1 Fusion Is Triggered by ROS1–JAK2–STAT3 Axis

**Figure 6:**
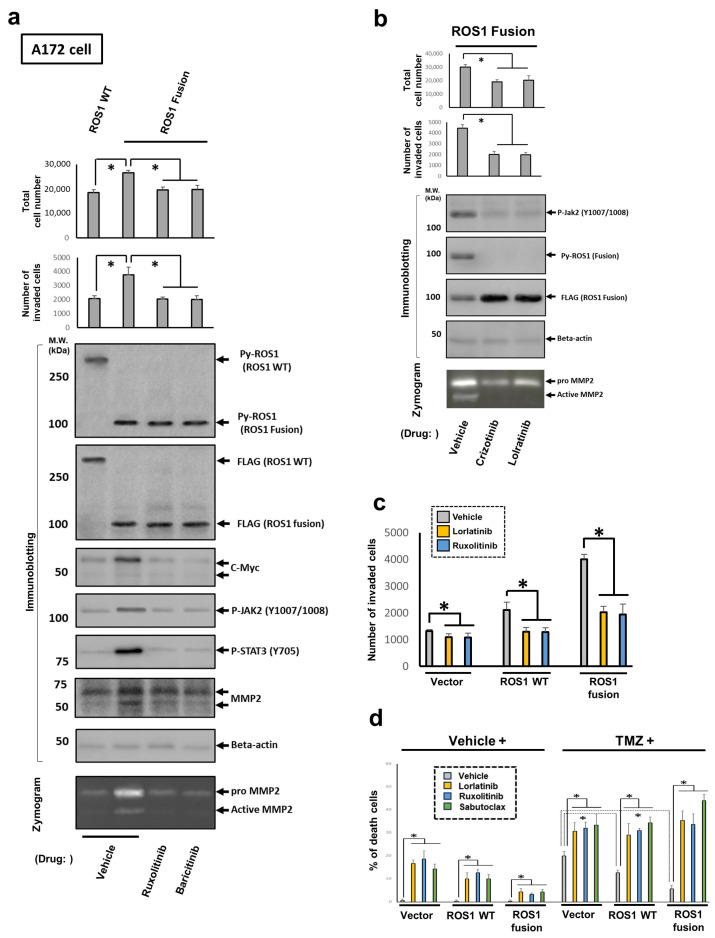
ROS1–JAK2–STAT3 axis-dependent upregulation of oncogenic properties of KLC1-ROS1 fusion expressed glioma cells compared with wild-type ROS1 expressed cells. (**a**) A172 cells stably expressing FLAG-tagged wild-type (WT) ROS1 or FLAG-tagged KLC1-ROS1 fusion, established in Figure 1a, were treated with the small-molecule JAK2 inhibitor ruxolitinib (1 μM) or baricitinib (1 μM), as well as vehicle (DMSO; control), as indicated. After 48 h, the total cell number was quantitated, and the cells were subjected to invasion assays. Cell lysates were analyzed using gelatin zymography to detect MMP2 activation. The primary antibody against beta actin was used for confirming the protein loading amount of each sample. * *p* > 0.01. (**b**) A172 cells stably expressing FLAG-tagged KLC1-ROS1 fusion were treated with the small-molecule ROS1 inhibitor crizotinib (500 nM), lorlatinib (100 nM), or vehicle (control), as indicated. After 48 h, cells were subjected to proliferation and invasion assays. Cell lysates were analyzed using gelatin zymography to detect MMP2 activation. The primary antibody against beta actin was used for controlling the amount of protein loading in each sample. * *p* > 0.01. The uncropped blots are shown in File S1. (**c**) A172 cells stably expressing FLAG-tagged WT ROS1, FLAG-tagged KLC1-ROS1 fusion, or empty vector, as seen in Figure 1a, were treated with the small-molecule ROS1 inhibitor lorlatinib (100 nM), the JAK2 inhibitor ruxolitinib (1 μM), and vehicle (DMSO; control), as indicated. After 48 h, the total cell number was quantitated, and the cells were subjected to invasion assays. * *p* > 0.01. (**d**) The A172 cells used in Figure 6c were treated with the small-molecule ROS1 inhibitor lorlatinib (100 nM), JAK2 inhibitor ruxolitinib (1 μM), Mcl-1 inhibitor sabutoclax (1 μM), or vehicle (DMSO; control) with vehicle (DMSO) or TMZ (150 μM), as indicated. After 72 h, the cells were subjected to cell death assay. * *p* > 0.01.

Finally, the molecular roles of the KLC1-ROS1 fusion–JAK2–STAT3 axis in the regulation of KLC1-ROS1 fusion-induced upregulated oncogenic properties in glioma cells were investigated. The cell proliferation and invasion, as well as the alteration of JAK-STAT pathway activation (assayed by immunoblotting) and the change in MMP2-mediated extracellular matrix (ECM) degradation (assayed by gelatin zymogram), in A172 cells stably expressing C’-terminal FLAG-tagged wild-type ROS1 and FLAG-tagged KLC1-ROS1 fusion, established in Figure 1a, treated with a vehicle or small-molecule JAK inhibitor, bacilitinib or ruxolitinib, under serum-reduced conditions were investigated as indicated in Figure 6a. The total cell number and cell invasion, JAK-STAT pathway activation, including downstream c-Myc and MMP2 expression, and MMP2-mediated ECM degradation of KLC1-ROS1 fusion-expressing cells were upregulated compared to those in wild-type ROS1 expressed cells (Figure 6a and Figure S1a); these upregulations in KLC1-ROS1 fusion-expressing cells was suppressed by treatment with each JAK inhibitor compared to the vehicle treatment (Figure 6a). In addition, the treatment of KLC1-ROS1 fusion-expressing cells with the small-molecule ROS1 inhibitors crizotinib or lorlatinib under serum-reduced conditions resulted in a decrease in the total cell number as well as the suppression of cell invasion, MMP2-mediated ECM degradation, and the activation of KLC1-ROS1 fusion and JAK2 (Figure 6b and Figure S1b). Moreover, an invasion assay of the A172 cells stably expressing empty vector, C’-terminal FLAG-tagged wild-type ROS1, and FLAG-tagged KLC1-ROS1 fusion, as seen in Figure 1a, under the treatment with lorlatinib or ruxolitinib, resulted in inhibition of the invasion of each cell when compared with the vehicle treatment, but the assay revealed a more efficient suppression of KLC1-ROS1 fusion expressed cell invasion compared with that of other cells (Figure 6c). Finally, to confirm the therapeutic efficacy of targeting the ROS1–JAK–STAT axis in KLC1-ROS1 fusion-harboring gliomas, the antitumor efficacies of lorlatinib, ruxolitinib, and the Mcl-1 inhibitor subtoclax, in combination with TMZ, were investigated. A172 cells stably expressing the empty vector, wild-type ROS1, or the KLC1-ROS1 fusion used in Figure 1b were treated with lorlatinib, ruxolitinib, or subtoclax in combination with TMZ, and the induction of cell death was assayed. Treatment with either lorlatinib, ruxolitinib, subtoclax, or TMZ triggered cell death in all of these cells but was less effective, especially in KLC1-ROS1 fusion-expressing cells (Figure 6d). When TMZ was combined with these three inhibitors, the efficiency of the cell death induction was exponentially enhanced, particularly in the KLC1-ROS1 fusion-expressing cells (Figure 6d). Based on these results, a contribution of the KLC1-ROS1 fusion-JAK2-STAT3-dependent machinery to enhance oncogenesis can be suggested, as well as the machinery being the therapeutic target of the KLC1-ROS1 fusion-harboring gliomas.

## 4. Discussion

Our study clarified the detailed oncogenic molecular machinery induced by KLC1-ROS1, a novel ROS1 fusion gene discovered in a glioma case. KLC1-ROS1 fusion specifically activated the JAK-STAT pathway among the major downstream signaling pathways of RTKs compared to wild-type ROS1. In previous reports on the intracellular localization of fusion proteins composed of the RTK activation loop, the activation of specific oncogenic signaling due to distinct subcellular localization of these fusion proteins dictated by the molecular characteristics of their fusion partners has been reported [37,38,39,40,41,42]. In the case of the ROS1 fusions, the SDC4-ROS1 and SLC34A2-ROS1 fusions reside in cytoplasmic endosomes and trigger MAPK activation [37]. CD74-ROS1 fusion is localized in the endoplasmic reticulum and activates RTK downstream pathways other than just MAPK signaling [37]. FIG-ROS1, primarily discovered in GBM cases, has been suggested to be predominantly localized in the Golgi apparatus, thereby conducting oncogenic signaling [42]. In the case of KLC1-ROS1 fusion, the N’N-terminal heptad repeat and TRP repeat domains of wild-type KLC1, which are required for the association of KLC1 with both the kinesin heavy chain and cytoplasmic cargo, respectively, are conserved in KLC1-ROS1 fusion (Figure 1a) [12]. In addition, as mentioned, the KLC1-ROS1 fusion protein did not possess any membrane-anchoring domains, unlike wild-type ROS1 (Figure 1a) [12]. Wild-type ROS1 is predominantly localized in the cytoplasm, particularly in the endoplasmic reticulum and mitochondria [43]. However, among the direct substrates of RTKs that conduct major RTK downstream signaling, JAKs have been shown to associate with and be activated by RTKs, mainly in the cytoplasm [44]. In the case of PI3K-Akt signaling, PI3K predominantly localizes and activates Akt at the inner side of the plasma membrane, and MAPK activator Ras families shuttle between the plasma membrane and cytoplasm like wild-type ROS1 [45]. Hence, from this past evidence, our result could be interpreted to indicate that KLC1-ROS1 fusion would mostly be localized in the cytoplasmic compartment just like JAK2 but unlike wild-type ROS1. Further investigations are needed to elucidate the mechanism by which KLC1-ROS1 fusion efficiently and specifically activates the JAK-STAT pathway compared to cytosolic wild-type ROS1.

In our data, KLC1-ROS1 fusion-expressing glioma cells efficiently exerted oncogenic properties compared with wild-type ROS1 expressed glioma cells, especially under serum-reduced conditions (Figure 5a), which was suggested to be triggered by the KLC1 domain-mediated constitutive serum-independent self-oligomerization of KLC1-ROS1 fusion. Since the fusion genes consisting of N’-terminal KLC1 domain and the C’-activation loop of RTK, other than ROS1, have already been reported [46], these fusions are supposed to exert upregulated oncogenicities by the same mechanism as well.

Our results revealed that inhibition of the JAK-STAT pathway affected the oncogenic properties of KLC1-ROS1 fusion expressed glioma cells (Figure 6a). These findings present an intriguing hypothesis: when a loss of the transmembrane domain and/or distinct intracellular localization due to the molecular features of the fusion partner of a ROS1 fusion is suggested, this ROS1 fusion may also govern distinct downstream molecular signaling and exert unique oncogenic properties compared with the wild-type ROS1. In connection with this hypothesis, it could be advocated that gliomas with such ROS1 fusions may be treated by drugs that target a pathway other than ROS1 activity. When addressing the treatment of gliomas that are highly refractory to various chemotherapeutic agents and where blood–brain barrier-mediated impaired drug delivery to the intracranial tumor tissue is an issue [47], multiple targets and their candidate drugs may increase the chances of overcoming these problems. Therefore, glioma cases with cytosolic ROS1 fusions could be proposed to possess greater opportunities to be given appropriate chemotherapy other than the current standard treatment using the alkylating agent temozolomide [48]. In the KLC1-ROS1 fusion-positive cases, from our data, the ROS1/JAK2/STAT3 axis inhibitors should be considered as alternative therapeutic agents. More importantly, these inhibitors demonstrated further effectiveness against glioma cells in combination with the first-line chemotherapeutic agent TMZ (Figure 6d), suggesting that the concurrent use of these inhibitors with each other or with other anticancer agents may become a novel therapeutic strategy against gliomas harboring KLC1-ROS1 fusion.

A limitation of this study is that we did not use pediatric low-grade (WHO grade II) glioma cells, similar to the original glioma case harboring *KLC1-ROS1* fusion that we previously reported [12]. To accurately depict the original pediatric glioma case harboring *KLC1-ROS1* fusion, proper glioma cell lines are crucial. In addition, we did not investigate whether wild-type KLC1 contributes to the oncogenicity of KLC1-ROS1 fusion-harboring glioma cells, although our current data suggest an essential role for the KLC1 domain in the activation of KLC1-ROS1 fusion. Since a recent study demonstrated the essential role of the upregulation of KLC1 expression in the oncogenesis of breast cancers [49], this point should be addressed in future studies.

## 5. Conclusions

Our results have demonstrated that the KLC1-ROS1 fusion-induced oncogenic mechanism is specifically driven by the ROS1–JAK2–STAT3 axis. It has also been suggested that drugs targeting this molecular machinery could be potent therapeutic agents for gliomas harboring KLC1-ROS1 fusion. Our results reemphasize the importance of detailed molecular investigations when a novel fusion gene is discovered, which may lead to the identification of alternative therapeutic targets.

## Data Availability

The data presented in this study are available in this article (and Supplementary Material).

## Supplementary Materials

The following supporting information can be downloaded at: https://www.mdpi.com/article/10.3390/cancers16010009/s1, Figure S1: The representative fluorescence images of Transwell based invasion assays; File S1: Full pictures of the Western blots.
