# Supplementary material for: KLC1-ROS1 Fusion Exerts Oncogenic Properties of Glioma Cells via Specific Activation of JAK-STAT Pathway
Source: Cancers (Basel). 2023 Dec 19;16(1):9. doi: 10.3390/cancers16010009 (PMC10778328; doi:10.3390/cancers16010009)
Supplement: Supplementary file 1 [file cancers-16-00009-s001.zip › Figure S1.pdf]

a

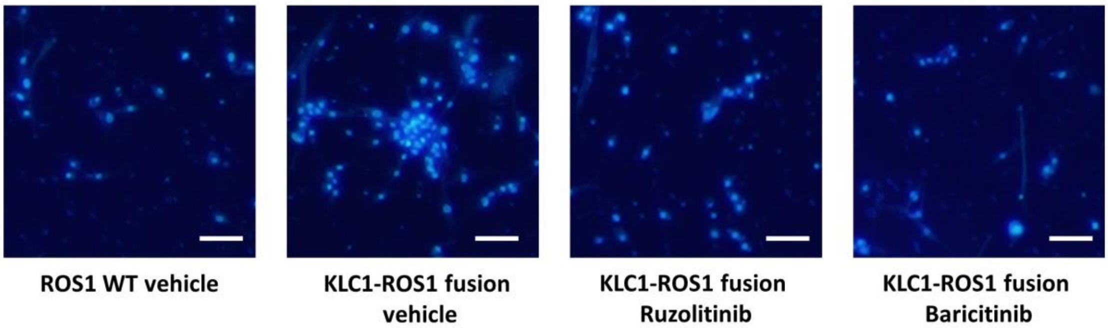

b

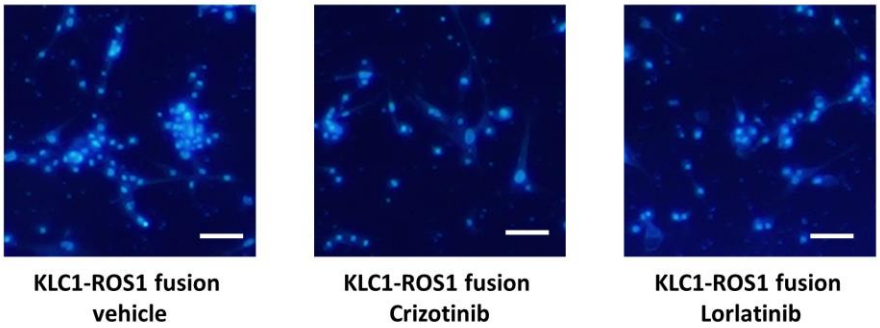

Figure S1. The representative fluorescence images of Transwell based invasion assays.

(**a**) The representative fluorescence microscope images of stained cell nuclei of invaded cells of figure 6a (bar, 50 μm). (**b**) The representative fluorescence microscope images of stained cell nuclei of invaded cells of figure 6b (bar, 50 μm).
