# Supplementary material for: KLC1-ROS1 Fusion Exerts Oncogenic Properties of Glioma Cells via Specific Activation of JAK-STAT Pathway
Source: Cancers (Basel). 2023 Dec 19;16(1):9. doi: 10.3390/cancers16010009 (PMC10778328; doi:10.3390/cancers16010009)

Figure 1a

A172 cell

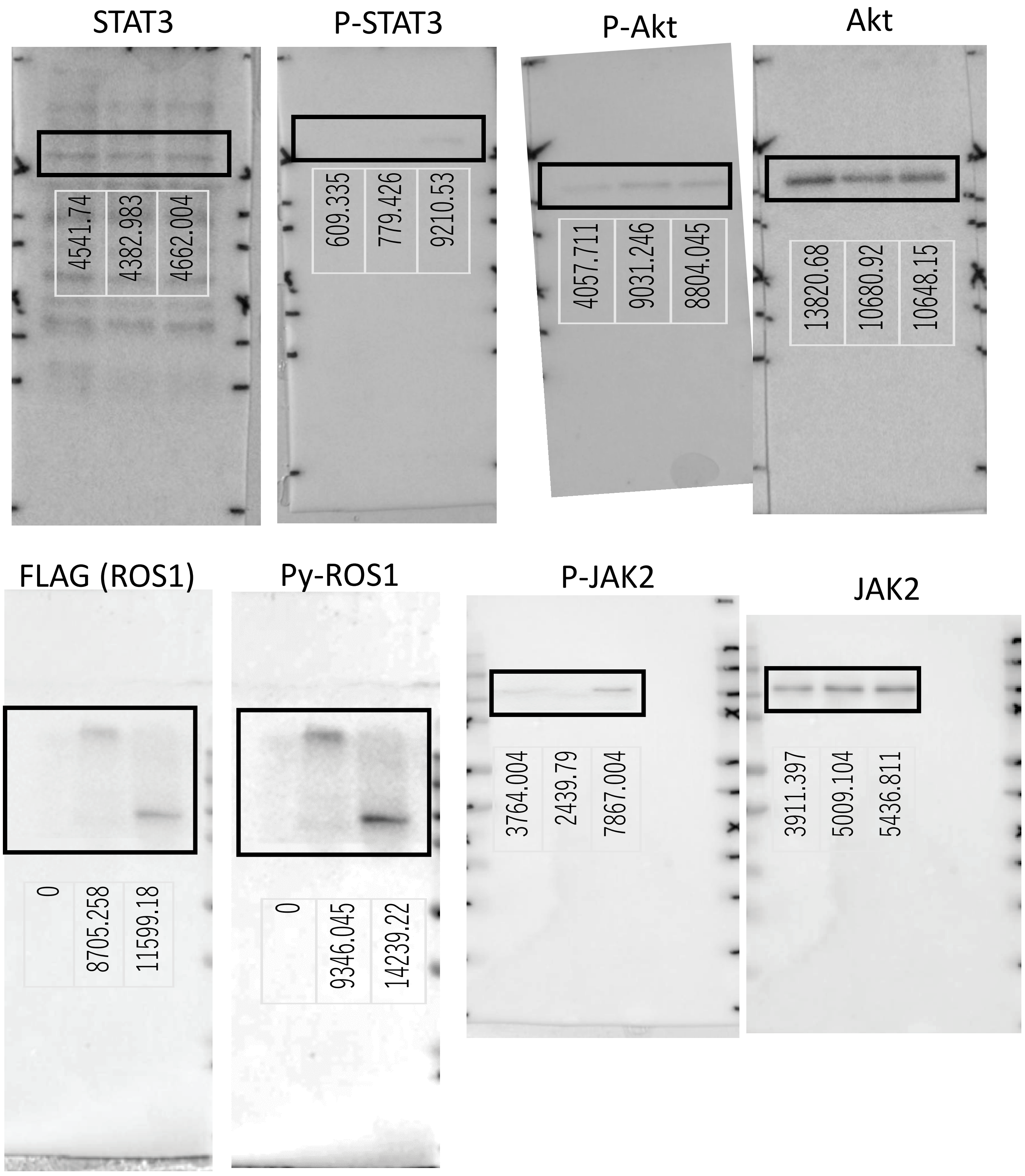

Figure 1a

A172 cell

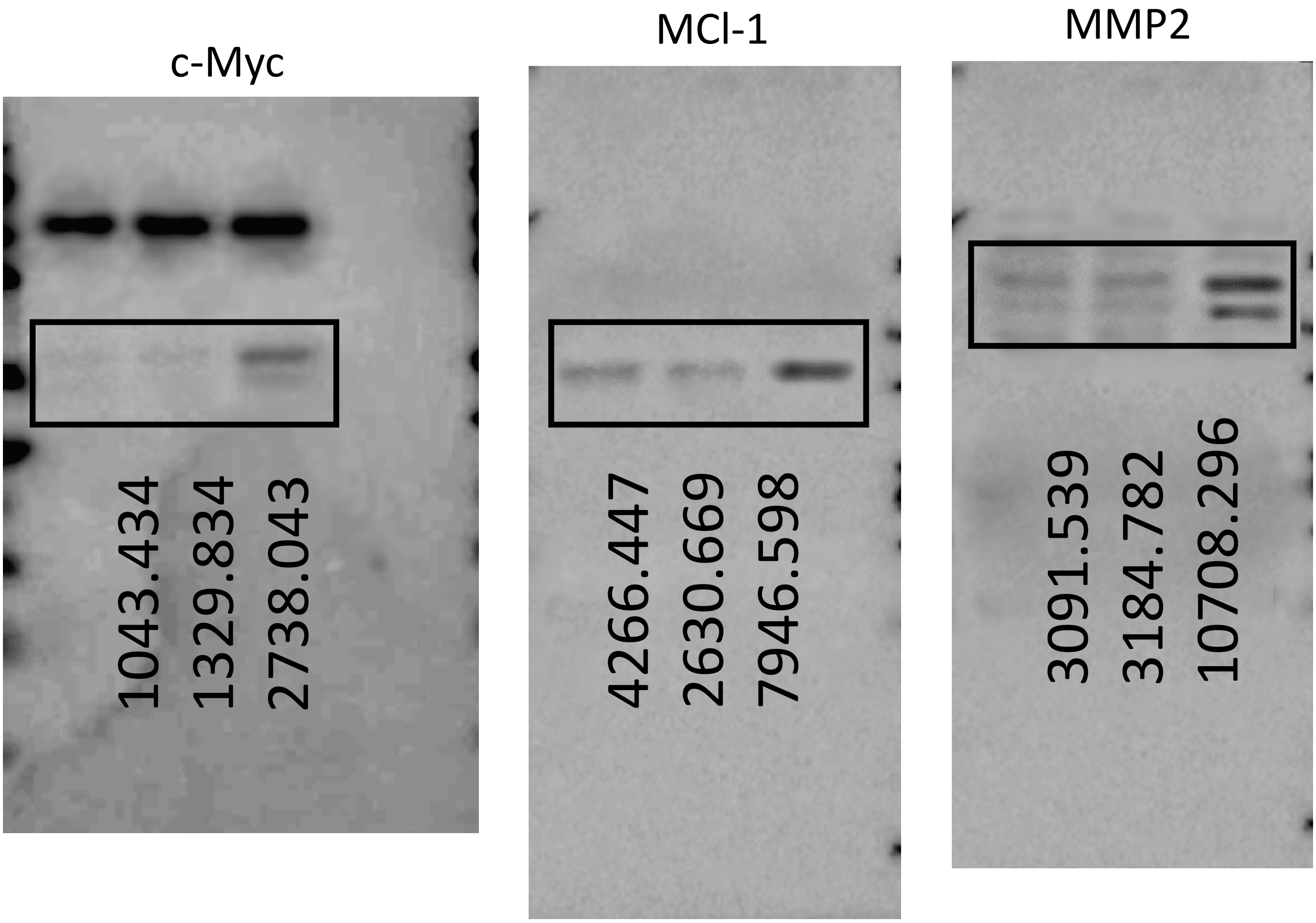

Figure 1a

A172 cell

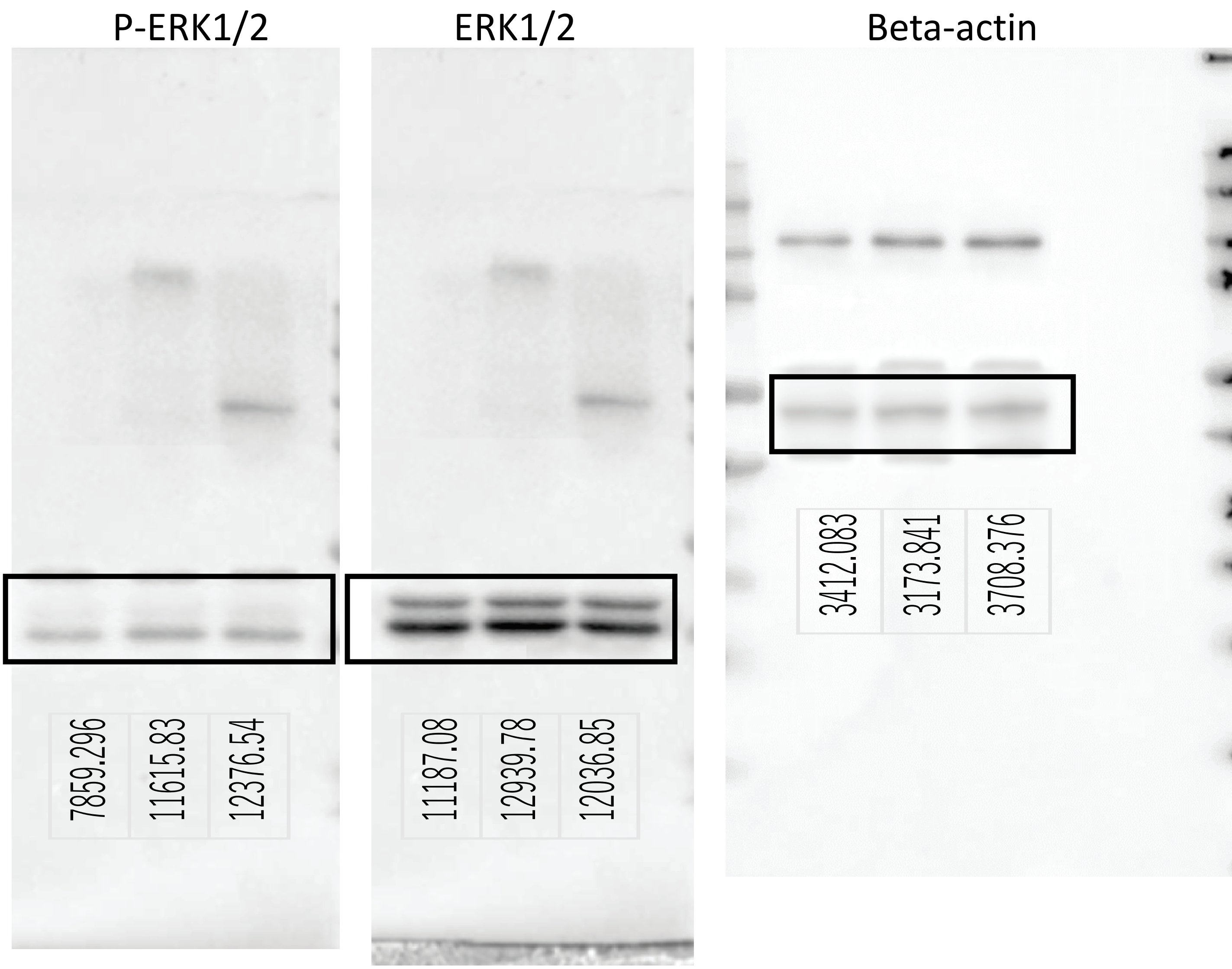

Figure 1b

U343MG cell

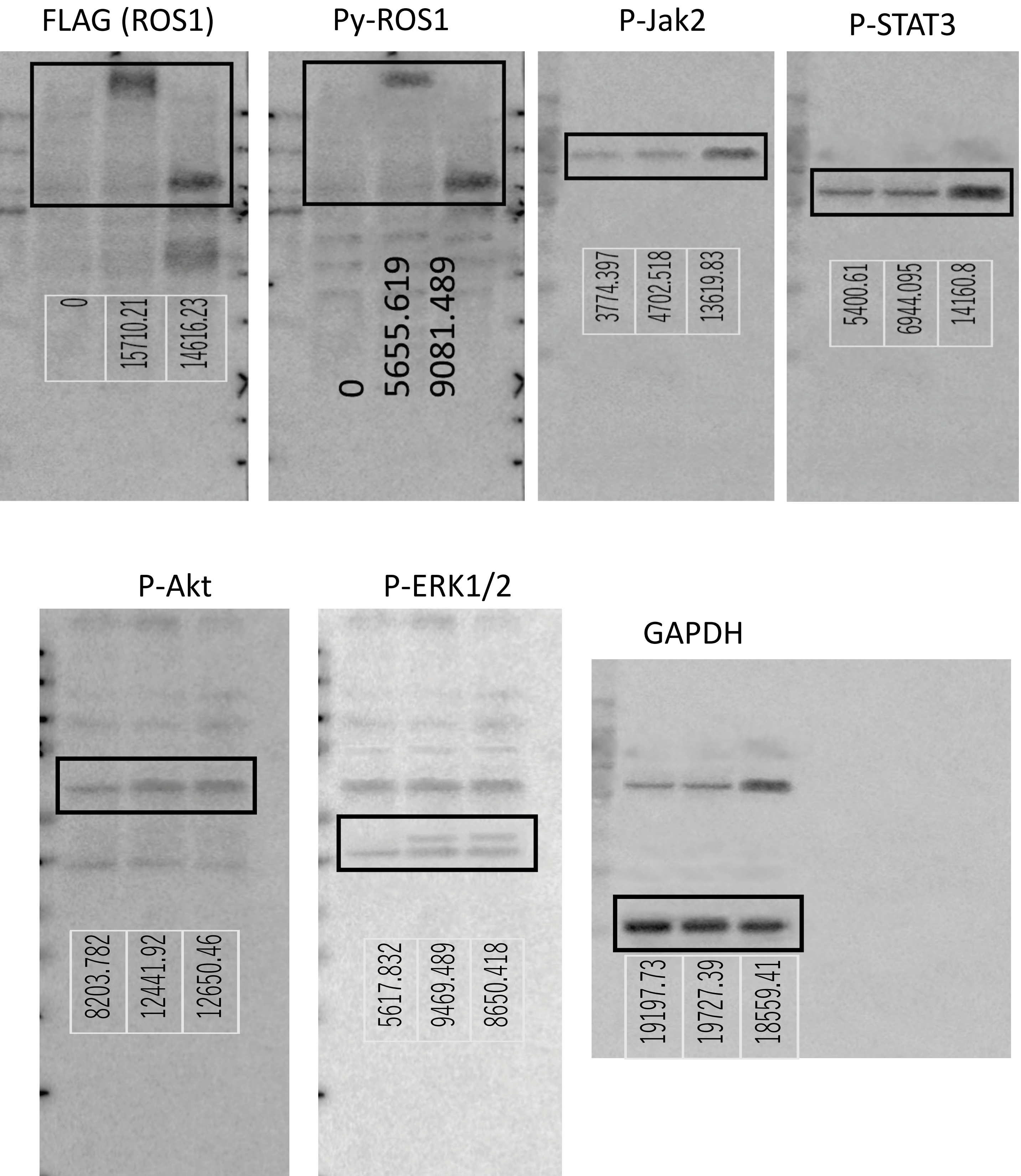

Figure 2a

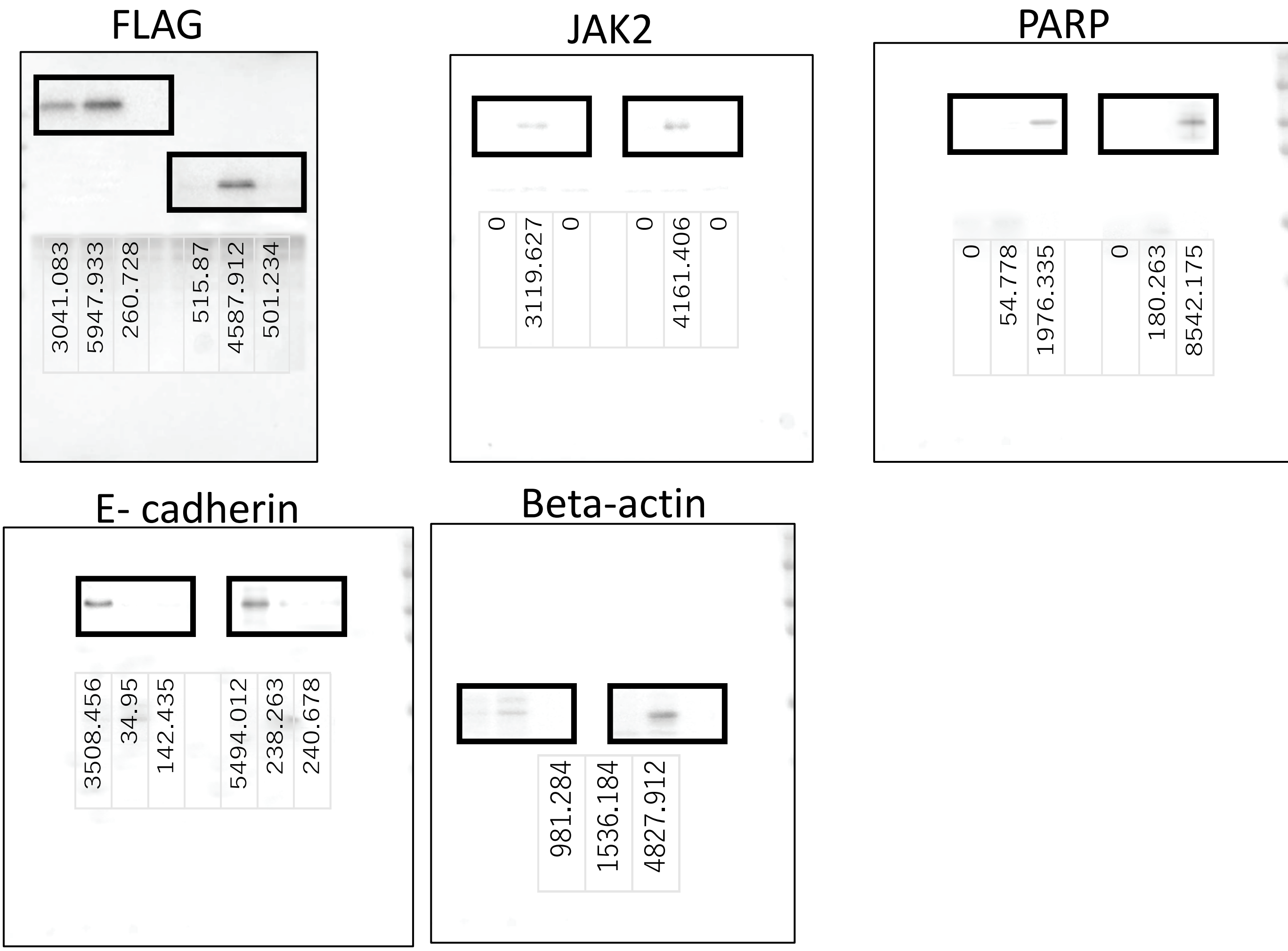
FLAG
3041.083
5947.933
260.728
515.87
4587.912
501.234
JAK2
0
3119.627
0
0
4161.406
0
PARP
0
54.778
1976.335
0
180.263
8542.175
E- cadherin
3508.456
34.95
142.435
5494.012
238.263
240.678
Beta-actin
981.284
1536.184
4827.912

Figure 2b

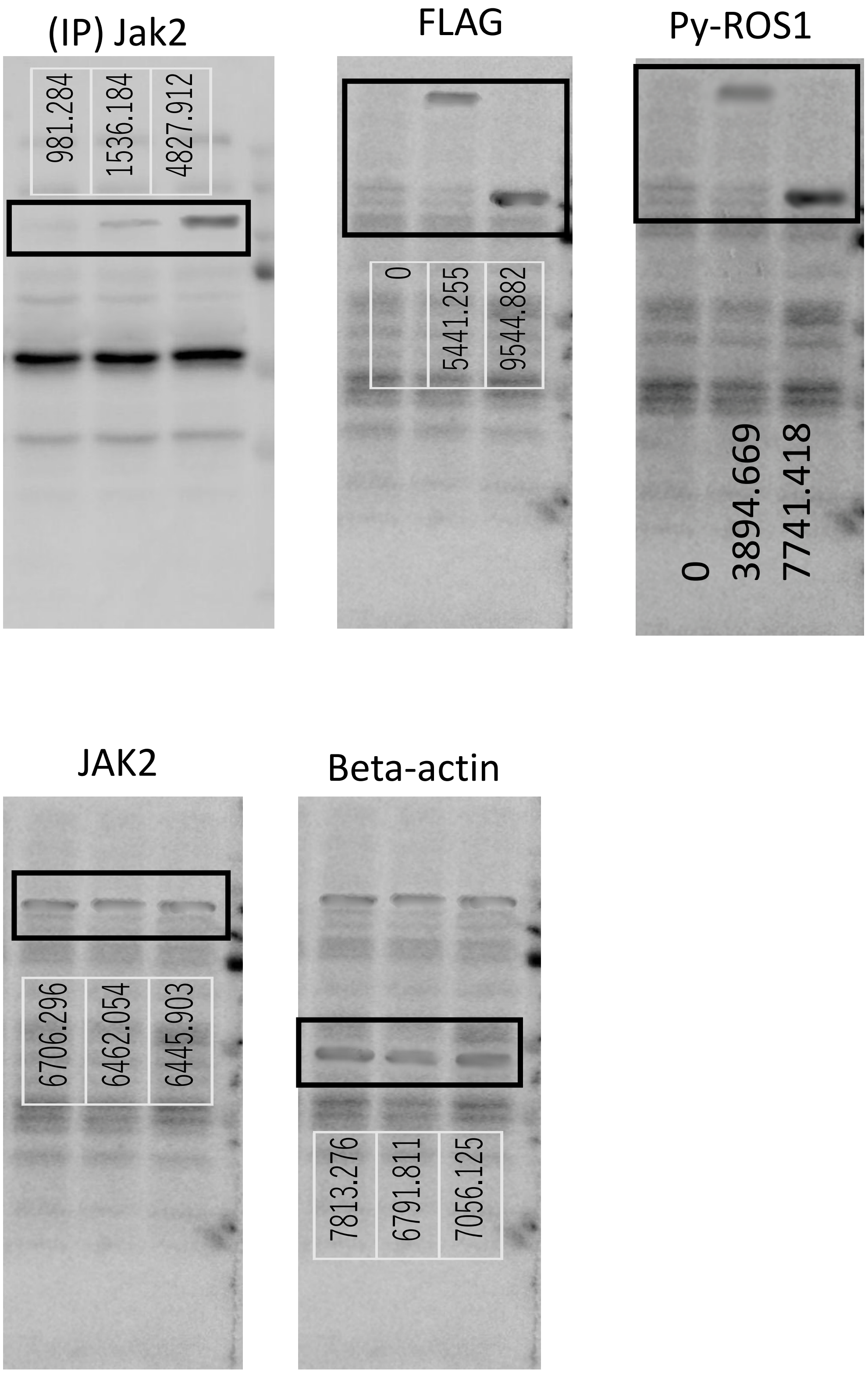
(IP) Jak2
981.284
1536.184
4827.912
FLAG
0
5441.255
9544.882
Py-ROS1
0
3894.669
7741.418
JAK2
6706.296
6462.054
6445.903
Beta-actin
7813.276
6791.811
7056.125

Figure 3b

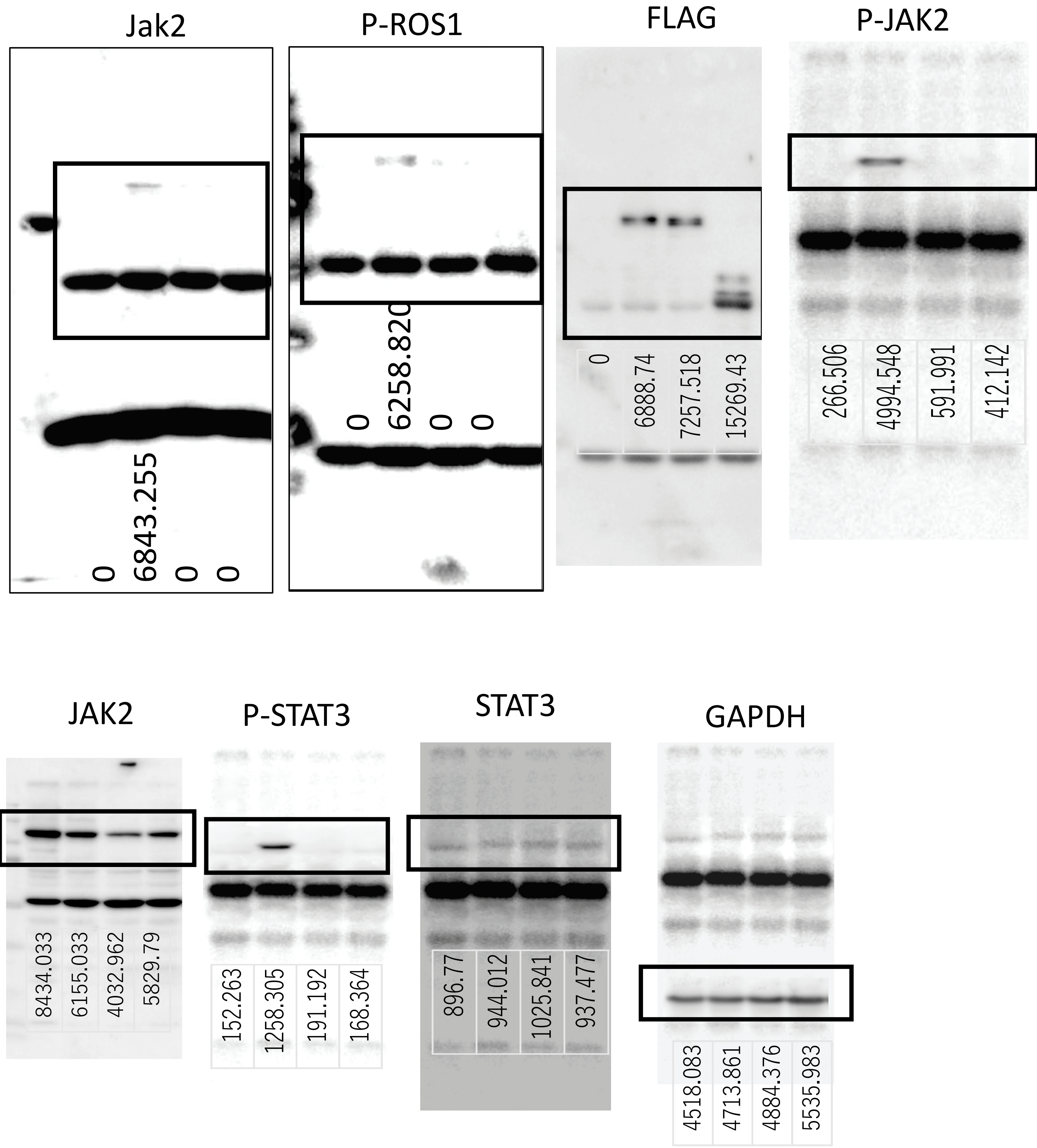
Jak2
0
6843.255
0
0
P-ROS1
0
6258.820
0
0
FLAG
0
6888.74
7257.518
15269.43
P-JAK2
266.506
4994.548
591.991
412.142
JAK2
8434.033
6155.033
4032.962
5829.79
P-STAT3
152.263
1258.305
191.192
168.364
STAT3
896.77
944.012
1025.841
937.477
GAPDH
4518.083
4713.861
4884.376
5535.983

Figure 3c

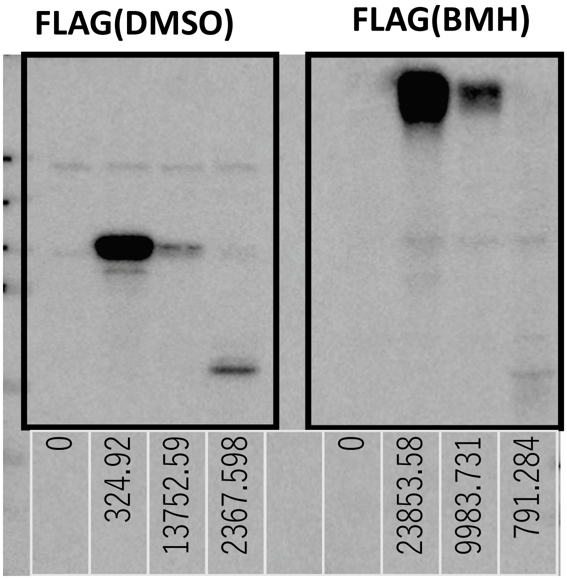

FLAG(DMSO)
FLAG(BMH)
0
324.92
13752.59
2367.598
0
23853.58
9983.731
791.284

Figure 4a

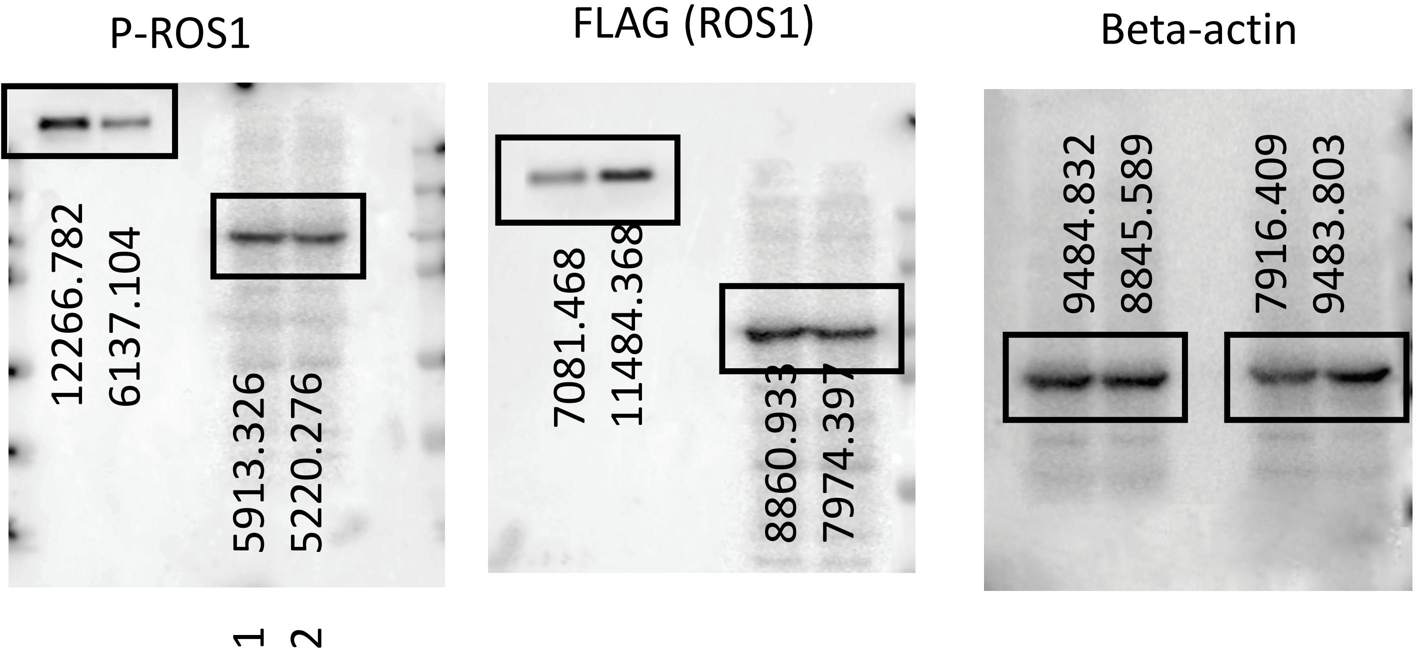

Figure 4b

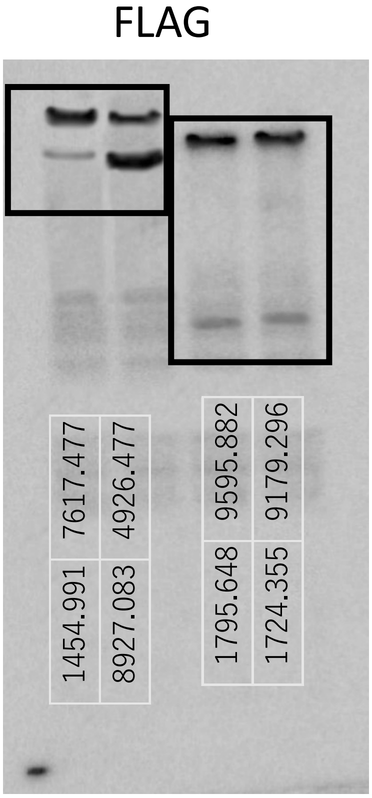

Figure 6a

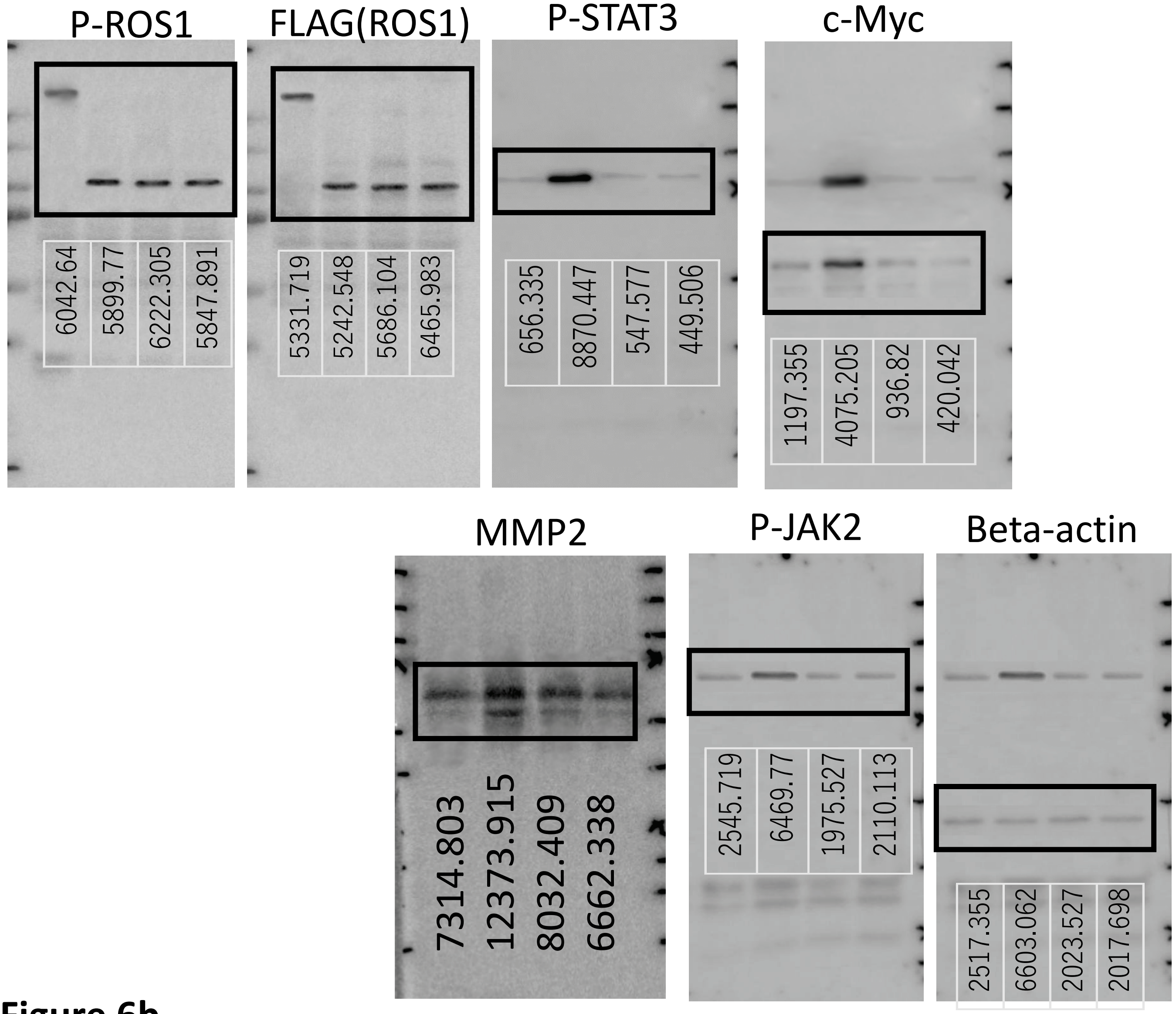

Figure 6b

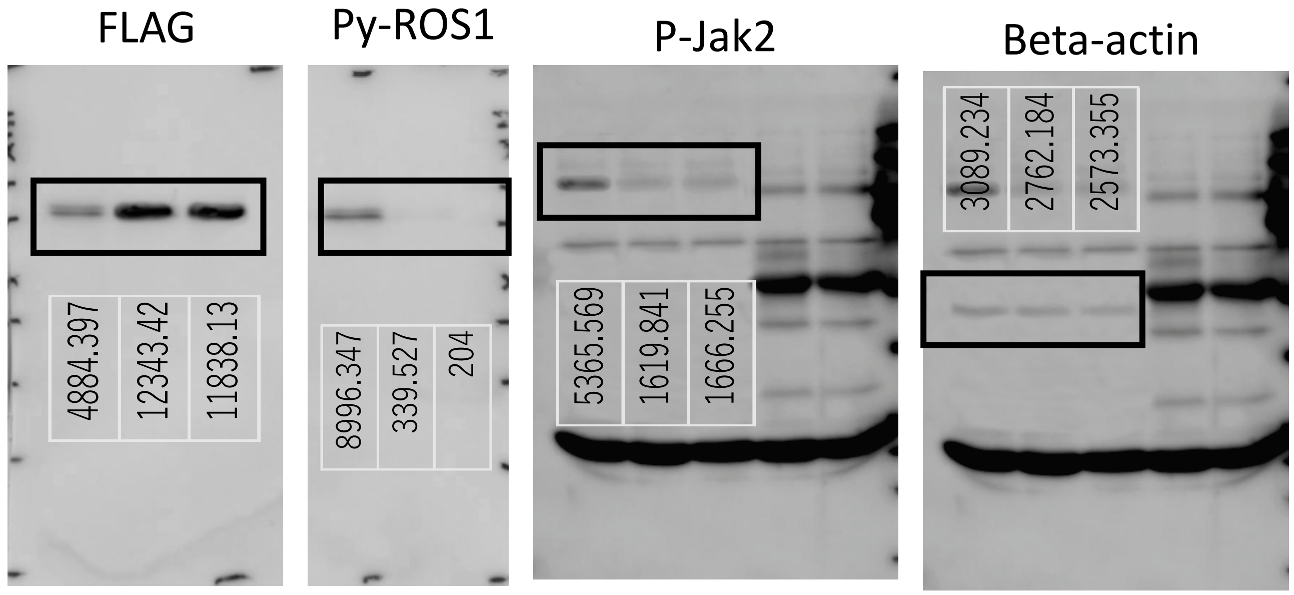

Figure 6a

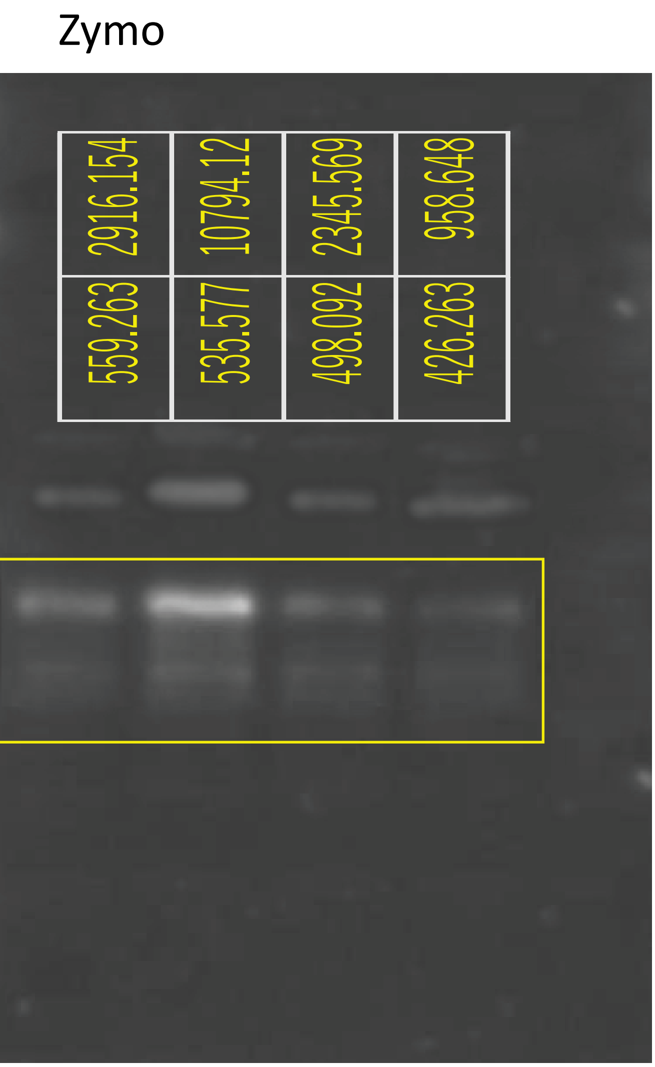

Figure 6b

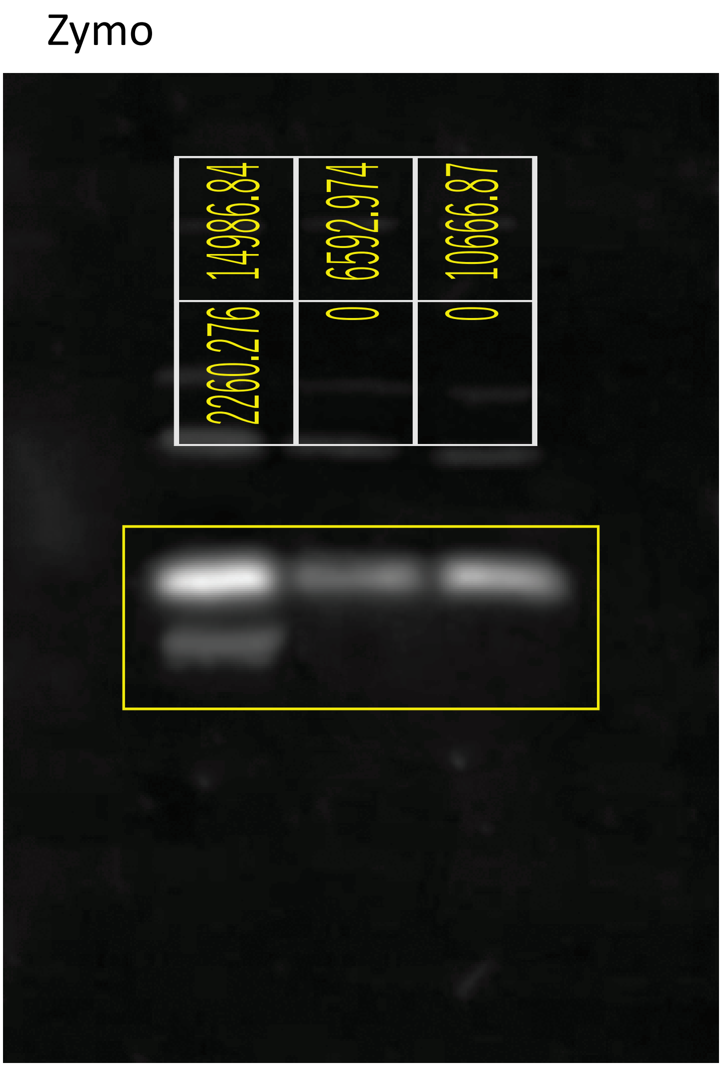

Supplement: Supplementary file 1 [file cancers-16-00009-s001.zip › File S1.pdf]
